# Plasma pentosidine as a useful biomarker of sarcopenia, low gait speed, and mortality in patients with cirrhosis

**DOI:** 10.3389/fmed.2023.1212899

**Published:** 2023-09-15

**Authors:** Chisato Saeki, Mitsuru Saito, Akihito Tsubota

**Affiliations:** ^1^Division of Gastroenterology and Hepatology, Department of Internal Medicine, The Jikei University School of Medicine, Tokyo, Japan; ^2^Division of Gastroenterology, Department of Internal Medicine, Fuji City General Hospital, Fuji, Shizuoka, Japan; ^3^Liver Disease Control Science, Graduate School of Organic Pathology and Therapeutics, The Jikei University School of Medicine, Tokyo, Japan; ^4^Department of Orthopedic Surgery, The Jikei University School of Medicine, Tokyo, Japan; ^5^Project Research Units, Research Center for Medical Science, The Jikei University School of Medicine, Tokyo, Japan

**Keywords:** pentosidine, cirrhosis, sarcopenia, low gait speed, prognosis

## Abstract

**Purpose:**

The accumulation of advanced glycation end products (AGEs) is associated with various diseases and age-related impairments, including loss of muscle mass and function. We investigated the association between plasma pentosidine, which is one of the AGEs, and sarcopenia, low gait speed, and mortality in patients with cirrhosis.

**Methods:**

This retrospective study divided 128 patients with cirrhosis into three groups by 25th and 75th quartiles of baseline plasma pentosidine levels: low (L)-, intermediate (I)-, and high (H)-pentosidine (Pen) groups. Sarcopenia was diagnosed following the Japan Society of Hepatology criteria. Low gait speed was defined as <0.8 m/s. The cumulative survival rates were compared between the three groups. Cox proportional hazards regression analysis was performed to identify independent factors associated with mortality.

**Results:**

Of the 128 patients, 40 (31.3%) and 34 (26.6%) had sarcopenia and low gait speed, respectively. The prevalence of sarcopenia and low gait speed significantly increased stepwise with increasing plasma pentosidine levels, with the highest in the H-Pen group (59.4% [19/32] and 56.3% [18/32], respectively) and lowest in the L-Pen group (18.8% [6/32] and 6.3% [2/32], respectively). Multivariate analysis identified plasma pentosidine levels as a significant and independent factor associated with sarcopenia (odds ratio [OR], 1.07; *p* = 0.036) and low gait speed (OR, 1.06; *p* = 0.036), with the cutoff levels of 0.0792 μg/mL (sensitivity/specificity, 0.600/0.773) and 0.0745 μg/mL (sensitivity/specificity, 0.735/0.691), respectively. The cumulative survival rates were significantly lower in the H-Pen group than in the L-Pen (hazard ratio [HR], 11.7; *p* = 0.001) and I-Pen (HR, 4.03; *p* < 0.001) groups. Plasma pentosidine levels were identified as a significant and independent prognostic factor (HR, 1.07; *p* < 0.001).

**Conclusion:**

Plasma pentosidine levels are associated with sarcopenia, low gait speed, and mortality and may serve as a useful surrogate biomarker for these clinical events in patients with cirrhosis.

## Introduction

Cirrhosis is caused by persistent hepatic inflammation and progressive liver fibrosis. It in turn causes various complications and is associated with high mortality (1). Cirrhosis progresses naturally in two stages: a compensated phase followed by a decompensated phase (with ascites, encephalopathy, jaundice, and variceal hemorrhage), eventually leading to liver failure (2). The 1-year mortality rate was four times higher in patients with decompensated cirrhosis than in those with compensated cirrhosis (3). Therefore, appropriate monitoring for disease progression and cirrhosis-related events and prognosis prediction are crucial for managing patients with cirrhosis.

Sarcopenia, which is a progressive and generalized loss of skeletal muscle mass and function, is one of the most common and critical complications in patients with cirrhosis (4). A meta-analysis of 36 studies reported that the pooled prevalence of sarcopenia was 33% in patients with cirrhosis (5). Recent studies revealed the existence of sarcopenia as a high-risk factor for cirrhosis-related events, such as infections, ascites, hepatic encephalopathy, and death (6–10). The pathophysiological mechanisms of sarcopenia in chronic liver disease are multifactorial and complex (11), i.e., aging, malnutrition, low insulin-like growth factor 1 (IGF-1, an anabolic hormone) and branched-chain amino acid levels, and elevated inflammatory cytokine levels cause an imbalance between protein synthesis and breakdown. In the aging population, low levels of vitamin D, folate, and albumin, high levels of C-reactive protein, older age, lower body mass index (BMI), and the existence of chronic diseases (including diabetes and renal dysfunction with albuminuria), which reflect malnutrition and inflammatory conditions, are closely associated with sarcopenia (12–14). Furthermore, sarcopenia, combined with cognitive impairment, increases 1-year mortality in older patients discharged from acute care hospitals (15). Therefore, the development and progression of sarcopenia should be evaluated from various viewpoints.

Advanced glycation end products (AGEs) are heterogeneous molecules of irreversible products formed by non-enzymatic reactions between reducing sugars and proteins/lipids/nucleic acids (known as the Maillard reaction) (16). Various factors, such as aging, hyperglycemia, smoking, excessive alcohol consumption, inflammation, and oxidative stress, promote the accumulation of AGEs, thereby promoting reactive oxygen species production and inflammatory cascades and changes in protein structure and function, cellular apoptosis and dysfunction, and various organ damage (17). Numerous studies have revealed that AGEs play a crucial role in the pathological conditions of various diseases, such as diabetes and its complications, poor bone quality and fractures, chronic kidney disease (CKD), neurodegenerative diseases, cardiovascular disease, and chronic liver disease (16, 18–23).

Pentosidine is one of the most widely studied AGEs. Its blood and urinary content correlate with the total amount of AGEs in tissues (such as bone and skin) and thus could be a representative marker for the total AGE content in the tissues (19, 24). Several studies have revealed that high serum or urinary pentosidine levels are associated with low muscle mass, low muscle strength, low gait speed, and sarcopenia in patients with diabetes and community-dwelling adults (25–27). Furthermore, high blood pentosidine levels are associated with mortality in patients with CKD or heart failure (28, 29). Our previous study of patients with chronic liver disease revealed increased plasma pentosidine levels stepwise as the liver disease progressed, and they were correlated with hepatic fibrosis marker and Child–Pugh scores (30). Conversely, liver functional reserve-related factors, such as total bilirubin, albumin, and prothrombin time, were associated with plasma pentosidine levels. Thus, plasma pentosidine levels may be useful in predicting sarcopenia, impaired liver functional reserve, and mortality in patients with cirrhosis.

This study aimed to investigate the relationship between plasma pentosidine levels, sarcopenia, and prognosis in patients with cirrhosis.

## Materials and methods

### Study participants

This retrospective study included 128 patients with cirrhosis who presented to the Jikei University School of Medicine (Tokyo, Japan) and Fuji City General Hospital (Shizuoka, Japan) between 2017 and 2020. The inclusion criteria were as follows: (1) the presence of cirrhosis and (2) available measurements of skeletal muscle mass index (SMI) using bioelectrical impedance analysis (InBody S10; InBody, Tokyo, Japan) and handgrip strength using a dynamometer (T.K.K5401 GRIP-D; Takei Scientific Instruments, Niigata, Japan). The exclusion criteria were as follows: (1) alcoholic cirrhosis with different characteristics from cirrhosis caused by other etiologies (e.g., low prevalence of sarcopenia in non-elderly patients despite advanced disease stage) (31); (2) pre-existence of uncontrollable malignancies, including hepatocellular carcinoma (HCC); and (3) pacemakers, implants, or refractory ascites due to the decreased reliability of bioelectrical impedance analysis. Cirrhosis was diagnosed based on laboratory tests and imaging/endoscopic findings, such as liver deformity with atrophy of the right hepatic lobe, nodular liver surface, ascites, and esophageal/gastric varices. The model for end-stage liver disease (MELD) score and the Child–Pugh classification were used to assess the liver functional reserve (32, 33). HCC diagnosis was made based on the HCC guidelines of the American Association for the Study of Liver Diseases (34). Patients with HCC were defined as those with viable HCC at study entry. Patients who underwent liver transplantation during the observation period were considered as death and censored cases. CKD was defined as an estimated glomerular filtration rate (eGFR) of <60 mL/min/1.73 m^2^ for at least 3 months (35). This study was conducted in accordance with the 2013 Declaration of Helsinki and was approved by the ethics committees of the Jikei University School of Medicine (approval number: 34-021) and Fuji City General Hospital (approval number: 279).

### Diagnosis of sarcopenia and low gait speed

The diagnosis of sarcopenia was made according to the revised sarcopenia criteria proposed by the Japan Society of Hepatology (36). In brief, sarcopenia was defined as low SMI (<7.0 kg/m^2^ for men and <5.7 kg/m^2^ for women) and low handgrip strength (<28 kg for men and < 18 kg for women). Gait speed was measured over a distance of 6 m, and low gait speed was defined as <0.8 m/s (37).

### Laboratory assessments

Serum total bilirubin, albumin, and creatinine were measured using standard laboratory methods. Mac-2 binding protein glycosylation isomer (M2BPGi, a hepatic fibrosis marker) was measured using a sandwich enzyme-linked immunosorbent assay (ELISA) with *Wisteria floribunda* lectin-recognizing carbohydrate chains (HISCL-2000i; Sysmex, Hyogo, Japan) and presented as a cut-off index (C.O.I.) calculated according to the manufacturer’s specified formula. Prothrombin time–international normalized ratio (PT–INR) was measured using a thromboplastin reagent (Coagpia PT-N; Sekisui Medical, Tokyo, Japan). The eGFR was calculated using the following formula: eGFR (mL/min/1.73 m^2^) = 194 × creatinine ^−1.094^ × age ^−0.287^ (×0.739 for women). Plasma pentosidine levels were measured using an ELISA (FSK pentosidine ELISA kit; Fushimi Pharmaceutical, Kagawa, Japan), as described elsewhere (30). Briefly, 50 μL of plasma samples were added to pronase and immediately incubated at 55°C for 1.5 h. After the enzyme reaction, they were heated in boiling water for 15 min to inactivate the enzyme. The pretreated plasma samples were incubated with pentosidine-specific rabbit antibody at 37°C for 1 h. After washing, peroxidase-labeled goat anti-rabbit IgG polyclonal antibody was added and re-incubated at room temperature for 1 h. A color-developing reagent was added to each well and the reaction was stopped after 10 min. Finally, the absorbance was measured at 450 nm (main wavelength) and 630 nm (reference wavelength).

### Classification based on plasma pentosidine levels

The median plasma pentosidine level of the study cohort was 0.0665 μg/mL (interquartile range, 0.0508–0.0954 μg/mL). Patients were classified into three groups based on the first and third quartiles: low-pentosidine (L-Pen) (pentosidine level: ≤0.0508 μg/mL); (2) intermediate-pentosidine (I-Pen) (pentosidine level: 0.0508–0.0954 μg/mL); and (3) high-pentosidine (H-Pen) (pentosidine level: ≥0.0954 μg/mL).

### Statistical analysis

Categorical variables and continuous variables are expressed as number (percentage) and median (interquartile range), respectively. The Shapiro–Wilk test was used to evaluate continuous variables for their normality of distribution. As a result, no continuous variables analyzed in this study were normally distributed; therefore, nonparametric tests were applied for all continuous variables. The chi-squared test for categorical variables and the Kruskal–Wallis test for continuous variables were used to assess between-group differences. The Cochran–Armitage trend test was used to evaluate trends between variables with two categories and variables with multiple categories. Univariate and multiple logistic regression analyses were performed to identify significant and independent factors associated with sarcopenia and low gait speed. The area under the receiver operating characteristic curve of pentosidine was constructed to determine the optimal cutoff values for predicting sarcopenia and low gait speed. The Kaplan–Meier method was used to estimate cumulative survival rates, and the log-rank test was used to compare between-group differences. Univariate and multiple Cox proportional hazards models were used to identify significant and independent factors associated with mortality. All statistical analyses were performed using SPSS Statistics version 27 (IBM Japan, Tokyo, Japan). A *p* value of <0.05 was considered statistically significant.

## Results

### Patient characteristics

Table 1 summarizes the baseline characteristics of the 128 study participants. This study cohort included 71 (55.5%) men, with a median age of all patients at 72.0 (63.0–78.0) years. The median plasma pentosidine level was 0.0665 (0.0508–0.0954) μg/mL. The median MELD score was 8.0 (7.0–10.0). The rates of Child–Pugh class B or C (B/C), sarcopenia, low gait speed, and HCC were 25.8% (33/128), 31.3% (40/128), 26.6% (34/128), and 21.1% (27/128), respectively.

**Table 1:** Baseline characteristics of the three groups classified according to the plasma pentosidine levels.

Variable	All patients	L-Pen	I-Pen	H-Pen	*p* value
Patients, *n* (%)	128	32 (25.0)	64 (50.0)	32 (25.0)	
Men/Women, *n* (%)	71(55.5)/57(44.5)	18 (56.3)/14 (43.8)	37(57.8)/27(42.2)	16 (50.0)/16 (50.0)	0.764
Age (years)	72.0 (63.0–78.0)	68.5 (59.3–73.5)	73.5 (64.0–80.0)	75.5 (63.5–78.0)	0.024
BMI (kg/m^2^)	23.8 (21.3–26.3)	25.4 (22.6–30.1)	24.1 (21.5–26.1)	22.3 (19.1–24.6)	0.004
Diabetes mellitus, *n* (%)	56 (43.8)	19 (59.4)	18 (28.1)	19 (59.4)	0.002
Chronic kidney disease, *n* (%)	66 (51.6)	16 (50.0)	30 (46.9)	21 (65.6)	0.212
Etiology					
HBV/HCV/other, n	17/61/50	4/9/19	9/35/20	4/17/11	0.090
Child-Pugh B/C, *n* (%)	33 (25.8)	3 (9.4)	10 (15.6)	20 (62.5)	< 0.001
MELD score	8.0 (7.0–10.0)	7.5 (7.0–9.0)	8.0 (7.0–9.0)	10.0 (8.0–13.8)	< 0.001
Total bilirubin (mg/dL)	0.8 (0.6–1.1)	0.9 (0.6–1.1)	0.8 (0.6–1.1)	0.8 (0.4–1.4)	0.870
Albumin (g/dL)	3.9 (3.5–4.3)	4.1 (3.6–4.4)	4.0 (3.6–4.3)	3.4 (2.9–3.7)	< 0.001
Prothrombin time INR	1.08 (1.00–1.18)	1.01 (1.00–1.13)	1.10 (1.00–1.16)	1.12 (1.05–1.26)	0.036
Creatinine (mg/dL)	0.89 (0.69–1.10)	0.80 (0.62–1.12)	0.90 (0.70–1.06)	1.05 (0.70–1.20)	0.217
eGFR (mL/min/1.73m^2^)	59 (47–72)	61 (50–87)	60 (51–69)	49 (38–72)	0.049
M2BPGi (C.O.I.)	2.94 (1.41–5.40)	1.56 (1.16–2.71)	2.86 (1.54–4.20)	6.22 (3.36–7.87)	< 0.001
Pentosidine (μg/mL)	0.0665 (0.0508–0.0954)	0.0421 (0.0377–0.0447)	0.0665 (0.0560–0.0768)	0.1299 (0.1087–0.1841)	< 0.001
SMI (kg/m^2^)					
All patients	6.55 (5.69–7.75)	7.72 (5.85–8.47)	6.64 (5.92–7.54)	6.01 (5.21–6.98)	0.007
Men	7.19 (6.48–8.24)	8.28 (7.57–8.99)	7.13 (6.51–8.02)	6.93 (6.25–7.18)	0.002
Women	5.81 (5.07–6.35)	5.54 (4.78–7.21)	5.96 (5.64–6.32)	5.31 (4.93–5.80)	0.154
Handgrip strength (kg)					
All patients	22.7 (16.8–32.2)	29.7 (16.0–36.9)	23.6 (18.9–34.9)	17.6 (13.3–24.5)	0.001
Men	30.5 (24.0–38.1)	35.2 (31.3–42.9)	31.6 (23.9–39.0)	23.8 (14.4–28.0)	< 0.001
Women	17.0 (14.2–21.3)	15.6 (14.5–21.1)	19.0 (14.2–21.9)	15.8 (13.1–17.7)	0.351
Sarcopenia, *n* (%)	40 (31.3)	6 (18.8)	15 (23.4)	19 (59.4)	< 0.001
Gait speed (m/s)	1.06 (0.79–1.21)	1.12 (0.99–1.25)	1.10 (0.87–1.24)	0.73 (0.55–1.04)	< 0.001
Low gait speed, *n* (%)	34 (26.6)	2 (6.3)	14 (21.9)	18 (56.3)	< 0.001
HCC, *n* (%)	27 (21.1)	3 (9.4)	13 (20.3)	11 (34.4)	0.096

Continuous variables are shown as median (interquartile range). Statistical analysis was performed using the chi-squared test or the Kruskal-Wallis test, as appropriate. BMI, body mass index; C.O.I., cut-off index; eGFR, estimated glomerular filtration rate; HBV, hepatitis B virus; HCC, hepatocellular carcinoma; HCV, hepatitis C virus; INR, international normalized ratio; M2BPGi, Mac-2 binding protein glycosylation isomer; MELD, model for end-stage liver disease; Pen, pentosidine; SMI, skeletal muscle mass index.

### Clinical characteristics of patients according to pentosidine levels

**Figure 1:**
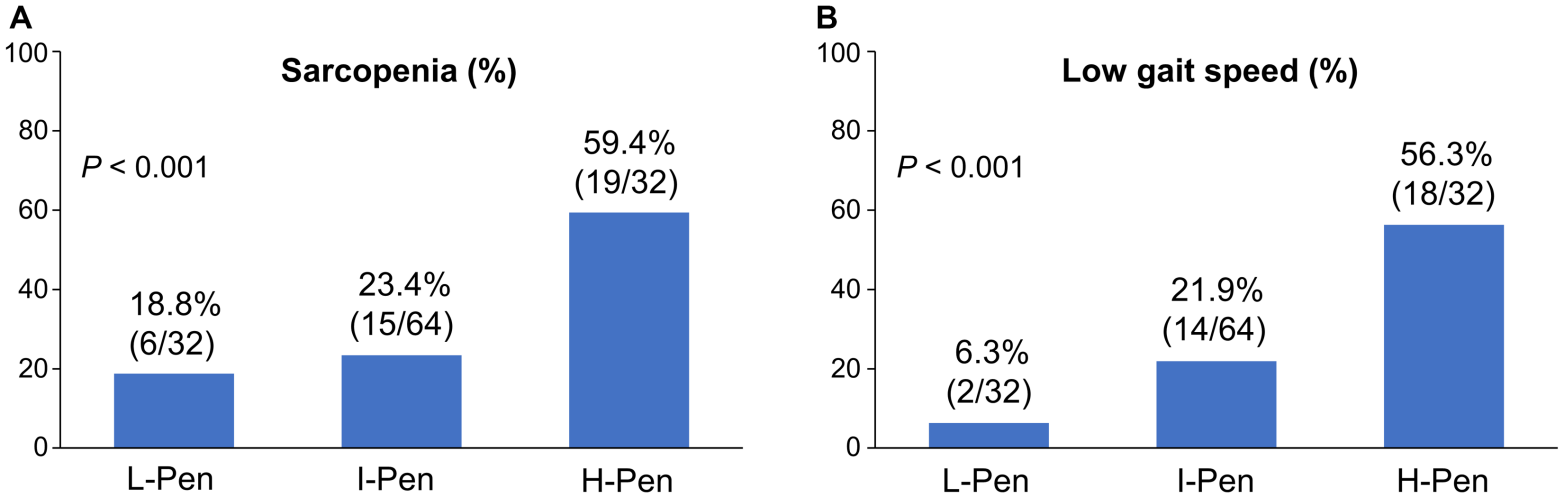
Comparison of the prevalence of sarcopenia and low gait speed between the three groups stratified according to baseline plasma pentosidine levels. **(A,B)** The prevalence of sarcopenia and low gait speed significantly increased stepwise with increasing plasma pentosidine levels (*p* < 0.001 for both). L-Pen, low-pentosidine group; I-Pen, intermediate-pentosidine group; H-Pen, high-pentosidine group.

The distributions of the L-Pen, I-Pen, and H-Pen groups were 25.0% (32/128), 50.0% (64/128), and 25.0% (32/128), respectively (Table 1). Age, BMI, diabetes mellitus prevalence, eGFR, M2BPGi, liver functional reserve-related factors (Child–Pugh class B/C, MELD score, albumin, and PT–INR), and sarcopenia-related factors (SMI, hand grip strength, and gait speed) significantly differed between the three groups. Of note, the prevalence of sarcopenia and low gait speed was highest in the H-Pen group (59.4% [19/32] and 56.3% [18/32], respectively) and lowest in the L-Pen group (18.8% [6/32] and 6.3% [2/32], respectively) (Figures 1A,B). The prevalence of these comorbidities significantly increased stepwise with increasing plasma pentosidine levels.

### Factors associated with sarcopenia and low gait speed

Six variables (age, BMI, diabetes mellitus, Child–Pugh class B/C, albumin, and pentosidine) were revealed as significant factors associated with sarcopenia in univariate analysis (Supplementary Table S1). Multivariate analysis identified advanced age (odds ratio [OR], 1.08; 95% confidence interval [CI], 1.02–1.14; *p* = 0.012), low BMI (OR, 0.73; 95% CI, 0.62–0.86; *p* < 0.001), and high pentosidine level (OR, 1.07; 95%CI, 1.01–1.15; *p* = 0.036) as significant and independent factors associated with sarcopenia in patients with cirrhosis (Table 2).

**Table 2:** Significant factors associated with sarcopenia in patients with cirrhosis.

Variable	Univariate		Multivariate	
	OR (95%CI)	*p* value	OR (95%CI)	*p* value
Age (years)	1.08 (1.03–1.13)	0.001	1.08 (1.02–1.14)	0.012
BMI (kg/m^2^)	0.69 (0.59–0.81)	< 0.001	0.73 (0.62–0.86)	< 0.001
Diabetes mellitus	2.63 (1.22–5.65)	0.014		
Child-Pugh B/C	2.33 (1.02–5.32)	0.044		
Albumin (g/dL)	0.53 (0.28–1.01)	0.054		
Pentosidine (x10^2^) (μg/mL)	1.10 (1.02–1.19)	0.012	1.07 (1.01–1.15)	0.036

BMI, body mass index; CI, confidence interval; OR, odds ratio.

Meanwhile, six variables (age, diabetes mellitus, Child–Pugh class B/C, MELD score, albumin, and pentosidine) were revealed as significant factors associated with low gait speed in univariate analysis (Supplementary Table S2). Multivariate analysis identified advanced age (OR, 1.10; 95%CI, 1.04–1.17; *p* = 0.001), Child–Pugh class B/C (OR, 4.31; 95%CI, 1.49–12.5; *p* = 0.007), and high plasma pentosidine level (OR, 1.06; 95%CI, 1.00–1.13; *p* = 0.036) as significant and independent factors associated with low gait speed (Table 3).

**Table 3:** Significant factors associated with low gait speed in patients with cirrhosis.

Variable	Univariate		Multivariate	
	OR (95%CI)	*p* value	OR (95%CI)	*p* value
Age (years)	1.07 (1.02–1.12)	0.007	1.10 (1.04–1.17)	0.001
Diabetes mellitus	1.95 (0.88–4.31)	0.099		
Child-Pugh B/C	4.03 (1.71–9.46)	0.001	4.31 (1.49–12.5)	0.007
MELD score	1.13 (0.98–1.30)	0.083		
Albumin (g/dL)	0.50 (0.26–0.99)	0.047		
Pentosidine (x10^2^) (μg/mL)	1.10 (1.02–1.18)	0.012	1.06 (1.00–1.13)	0.036

CI, confidence interval; OR, odds ratio.

### Optimal cutoff plasma pentosidine levels for predicting sarcopenia and low gait speed

**Figure 2:**
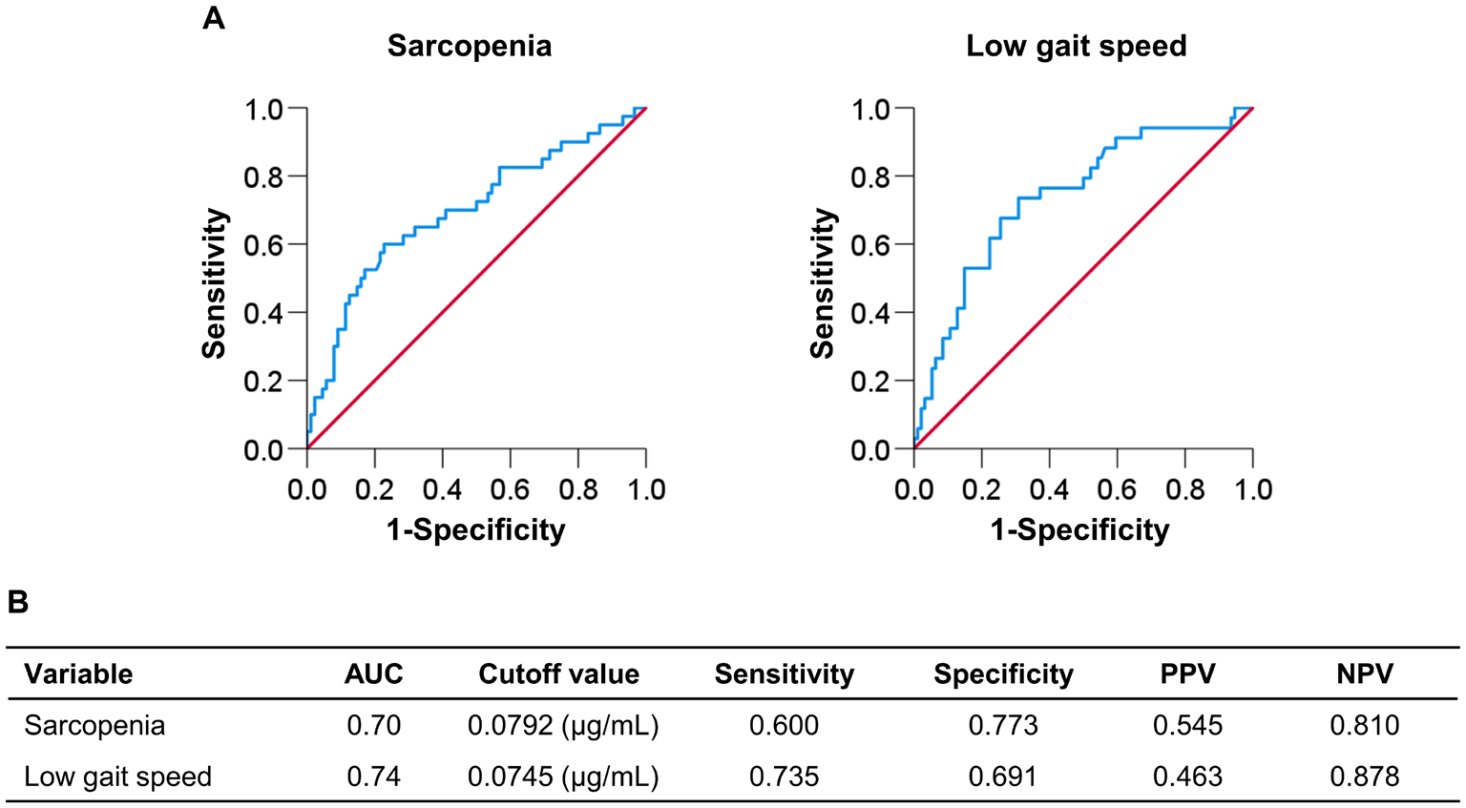
Receiver operating characteristic curve analysis of plasma pentosidine levels for the prediction of sarcopenia and low gait speed. **(A,B)** The cutoff value for predicting sarcopenia was 0.0792 μg/mL, with an area under the curve (AUC), sensitivity, and specificity of 0.70, 0.600, and 0.773, respectively. The cutoff value for predicting low gait speed was 0.0745 μg/mL, with an AUC, sensitivity, and specificity of 0.74, 0.735, and 0.691, respectively. PPV, positive predictive value; NPV, negative predictive value.

Figure 2 shows the cutoff values and diagnostic performance of plasma pentosidine levels for predicting sarcopenia and low gait speed. The cutoff plasma pentosidine levels for predicting sarcopenia and low gait speed were 0.0792 μg/mL (area under the curve [AUC], 0.70; sensitivity/specificity, 0.600/0.773) and 0.0745 μg/mL (AUC, 0.74; sensitivity/specificity, 0.735/0.691), respectively.

### Comparison of cumulative survival rates according to plasma pentosidine levels

**Figure 3:**
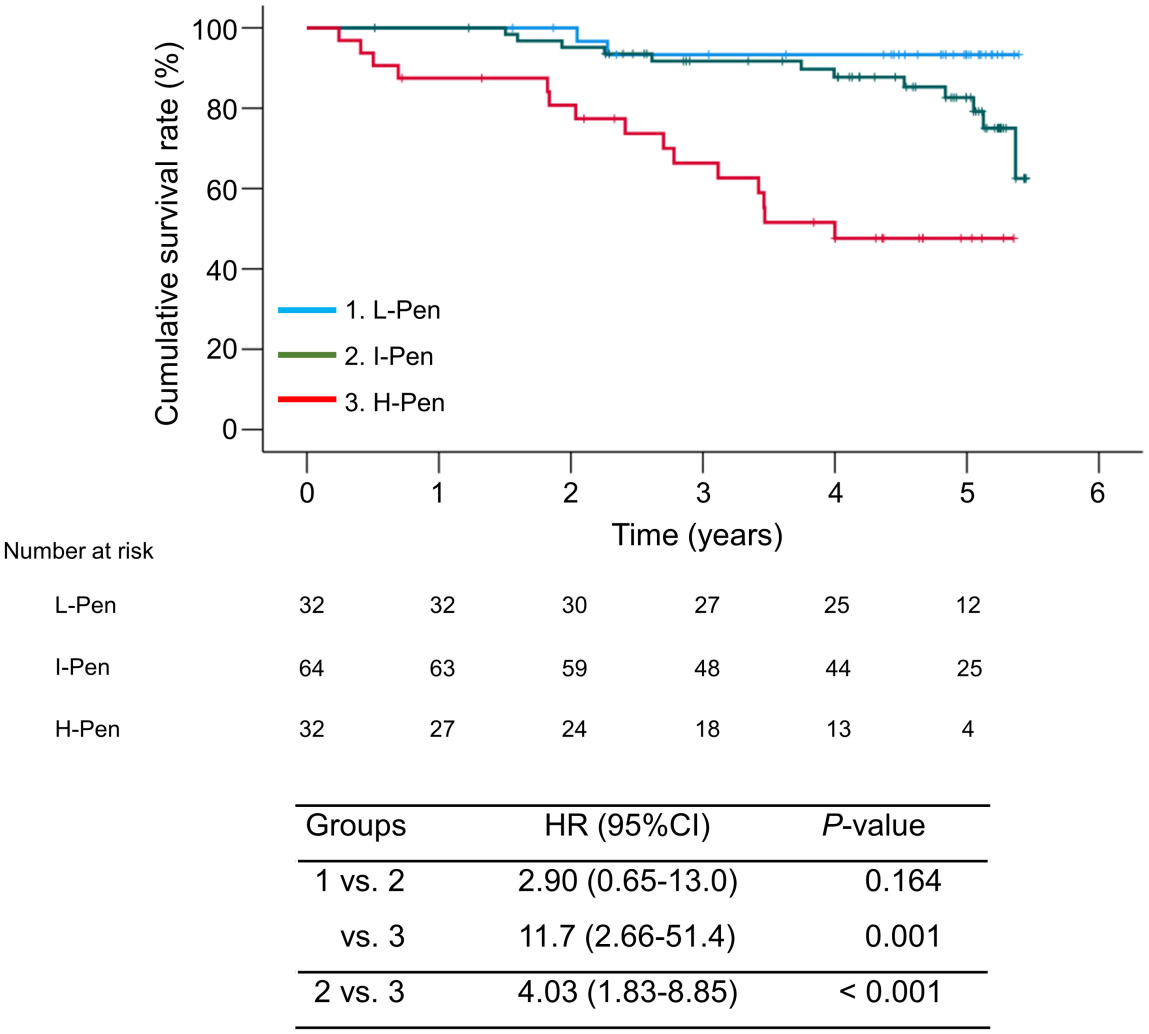
Comparison of the cumulative survival rates between the three groups stratified according to baseline plasma pentosidine levels. The cumulative survival rates were significantly lower in the high-pentosidine (H-Pen) group than in the low-pentosidine (L-Pen) and intermediate-pentosidine (I-Pen) groups (*p* = 0.001 and <0.001, respectively). Bands above and below polygonal lines denote 95% confidence interval.

The median observation period was 54.3 (33.6–61.1) months. During the observation period, 29 (22.7%) patients died of liver disease-related events (liver failure, *n* = 12; HCC, *n* = 11; liver transplantation, *n* = 2; rupture of esophageal varices, *n* = 4). The 1-, 3-, and 5-year cumulative survival rates were 87.5, 66.3, and 47.6% in the H-Pen group; 100, 91.7, and 82.7% in the I-Pen group; and 100, 93.3, and 93.3% in the L-Pen group, respectively. The cumulative survival rates were significantly lower in the H-Pen group than in the L-Pen (hazard ratio [HR], 11.7; 95%CI, 2.66–51.4; *p* = 0.001) and I-Pen (HR, 4.03; 95%CI, 1.83–8.85; *p* < 0.001) groups (Figure 3).

### Factors associated with mortality

Child–Pugh class B/C, MELD score, M2BPGi, pentosidine, sarcopenia, low gait speed, and HCC were significant factors associated with mortality in univariate analysis (Supplementary Table S3). Cox proportional hazards regression analysis identified high pentosidine level (HR, 1.07; 95% CI, 1.04–1.10; *p* < 0.001), low gait speed (HR, 3.58; 95% CI, 1.66–7.72; *p* < 0.001), and HCC (HR, 4.50; 95% CI, 2.05–9.86; *p* < 0.001) as significant and independent prognostic factors in patients with cirrhosis (Table 4).

**Table 4:** Significant factors associated with mortality in patients with cirrhosis.

Variable	Univariate		Multivariate	
	HR (95%CI)	*p* value	HR (95%CI)	*p* value
Child-Pugh B/C	5.47 (2.56–11.7)	< 0.001		
MELD score	1.22 (1.11–1.35)	< 0.001		
M2BPGi (C.O.I.)	1.06 (0.10–1.12)	0.073		
Pentosidine (x10^2^) (μg/mL)	1.09 (1.06–1.12)	< 0.001	1.07 (1.04–1.10)	< 0.001
Sarcopenia	2.49 (1.20–5.17)	0.015		
Low gait speed	3.62 (1.72–7.61)	< 0.001	3.58 (1.66–7.72)	< 0.001
HCC	4.69 (2.22–9.90)	< 0.001	4.50 (2.05–9.86)	< 0.001

CI, confidence interval; C.O.I., cut-off index; HCC, hepatocellular carcinoma; HR, hazard ratio; Mac-2 binding protein glycosylation isomer; MELD, model for end-stage liver disease.

## Discussion

AGEs, which are harmful compounds of irreversible non-enzymatic glycation products, play a crucial role in various diseases pathogenesis, including musculoskeletal diseases (16, 18–23). Several studies suggested that the accumulation of AGEs in the muscle induces loss of muscle mass and strength, and increased levels of blood AGEs are associated with sarcopenia and low gait speed (25–27, 38). Furthermore, high levels of plasma/serum AGEs and their precursors were associated with impaired liver functional reserve, leading to poor prognosis (30, 39–41). The present study investigated the relationship between plasma pentosidine levels (a representative marker of total AGEs), sarcopenia or low gait speed, and mortality in patients with cirrhosis. We found the highest prevalence of sarcopenia and low gait speed in the H-Pen group and the lowest in the L-Pen group. These comorbidities had significantly increased prevalence in a stepwise manner with increasing plasma pentosidine levels. Additionally, multivariate analysis identified plasma pentosidine levels as an independent factor associated with sarcopenia and low gait speed. One study of community-dwelling elderly adults revealed significant and negative associations between the urinary pentosidine level and handgrip strength and gait speed (26). Another study of middle-aged and elderly men with diabetes mellitus revealed serum pentosidine level as an independent risk factor of sarcopenia, and the prevalence of sarcopenia increased stepwise with an increased quartile of serum pentosidine levels (27). The other study of postmenopausal women with diabetes mellitus revealed increased serum pentosidine and decreased serum IGF-1 levels to be independently associated with loss of muscle mass (25). Further, older women with high serum carboxymethyl-lysine (CML) levels, which is one of the AGEs, had an increased risk of severe walking disability (i.e., inability to walk or ability to walk only at a speed of <0.4 m/s) (42). An *in vivo* study of mice with streptozotocin-induced diabetes revealed that AGE accumulation in hind limb muscles resulted in a decrease in muscle mass, muscle endurance, and regenerative capacity (38). In contrast, the administration of alagebrium chloride, which is an inhibitor of AGEs, ameliorated muscle atrophy and muscle regenerative capacity. An *in vitro* revealed that IGF-1 promoted the differentiation of myoblastic C2C12 cells, whereas AGEs decreased endogenous expression levels of IGF-1 mRNA and inhibited IGF-1-induced Akt activation, thereby increasing apoptosis and suppressing myogenic differentiation in C2C12 cells (43). IGF-1 is primarily synthesized in hepatocytes, and its levels are reduced in patients with advanced liver disease (11). Our previous study revealed an association between low serum IGF-1 levels and sarcopenia and low gait speed in patients with cirrhosis (44). Collectively, these reports indicate that increased plasma/serum AGE levels and decreased IGF-1 levels due to impaired liver functional reserve and accumulated AGEs in the muscle tissues are associated with sarcopenia and impaired physical performance and may be a predictor of the development and progression of these comorbidities in clinical settings. To our best knowledge, the present study is the first to focus on and clarify the relationship between plasma pentosidine (representative of AGEs) levels and sarcopenia or low gait speed in patients with cirrhosis, because of the few related literature.

This study revealed decreased survival rates stepwise with increasing plasma pentosidine levels, which were a significant and independent prognostic factor. One study of patients with CKD revealed a positive association between plasma pentosidine levels and oxidative stress and inflammation markers and plasma pentosidine levels as an independent predictor of mortality (28). Further, a study of patients with heart failure reported an independent association between high serum pentosidine levels and cardiac events (defined as cardiac death and rehospitalization due to heart failure exacerbation) (29). These findings indicate that plasma/serum pentosidine levels may be useful for predicting prognosis in disease conditions with increased AGEs.

Circulating AGEs are eliminated via the scavenger receptors of sinusoidal Kupffer and endothelial cells in the liver (45). Accordingly, liver disease progression causes a decline in the clearance of circulating molecules due to incomplete subendothelial basal membrane formation (40). Indeed, plasma/serum AGE levels correlate with liver fibrosis markers and Child-Pugh scores (30, 39–41). Intriguingly, liver transplantation decreases by approximately 50% in the CML level within 3 months after transplantation, despite persistent renal impairment, thereby highlighting the crucial role of the liver in AGE elimination (39). Thus, more advanced liver function impairment may facilitate the accumulation of AGEs in patients with cirrhosis. In turn, the accumulated AGEs will increase oxidative stress and inflammation, thereby further deteriorating liver injury and fibrosis (17). A clinical study reported significantly higher serum AGE levels in patients with HCC than in those with non-alcoholic steatohepatitis and control subjects (46). Furthermore, high serum AGE levels were independently associated with an increased risk of HCC, particularly in patients with hepatitis C-related cirrhosis (47). *In vitro* studies revealed that AGEs and related-receptors were associate with angiogenesis and tumor differentiation of HCC (48, 49). Hence, high AGE levels may be involved in liver fibrosis progression, liver function impairment, and HCC tumorigenesis, and consequently, worsen prognosis in patients with chronic liver disease.

This study has some limitations. First, we were unable to assess the accumulation of AGEs in muscle tissues. Second, we did not investigate the patient’s lifestyle, such as dietary intake or physical exercise, which may impact the development and progression of sarcopenia. Finally, this was a retrospective, small-group study conducted at two medical centers; therefore, prospective, large-scale, multicenter studies are required to confirm the obtained findings.

## Conclusion

In conclusion, the plasma pentosidine level was significantly and independently associated with sarcopenia, low gait speed, and mortality. Therefore, it may serve as a surrogate biomarker for these clinical events in patients with cirrhosis.

## Data availability statement

The raw data supporting the conclusions of this article will be made available by the authors, without undue reservation.

## Ethics statement

The studies involving humans were approved by the Jikei University School of Medicine (approval number: 34-021) and Fuji City General Hospital (approval number: 279). The studies were conducted in accordance with the local legislation and institutional requirements. The ethics committee/institutional review board waived the requirement of written informed consent for participation from the participants or the participants’ legal guardians/next of kin because of the retrospective nature of the study.

## Author contributions

CS and MS participated in the conception and design of the study. CS and AT acquired, analyzed and interpreted the data, and drafted the manuscript. MS and AT interpreted the data and revised the manuscript. AT substantively revised and completed the manuscript. All authors read and approved the final version of the manuscript.

## Conflict of interest

The authors declare that the research was conducted in the absence of any commercial or financial relationships that could be construed as a potential conflict of interest.

## Publisher’s note

All claims expressed in this article are solely those of the authors and do not necessarily represent those of their affiliated organizations, or those of the publisher, the editors and the reviewers. Any product that may be evaluated in this article, or claim that may be made by its manufacturer, is not guaranteed or endorsed by the publisher.
