# Supplementary material for: Plasma pentosidine as a useful biomarker of sarcopenia, low gait speed, and mortality in patients with cirrhosis
Source: Front Med (Lausanne). 2023 Sep 15;10:1212899. doi: 10.3389/fmed.2023.1212899 (PMC10541311; doi:10.3389/fmed.2023.1212899)
Supplement: Supplementary file 1 [file Table_1.docx]

**Table S1. Univariate analysis of factors associated with sarcopenia**

| Variable | OR (95% CI) | *p* value |
| --- | --- | --- |
| Gender (Man) | 0.54(0.25–1.15) | 0.110 |
| Age (years) | 1.08(1.03–1.13) | 0.001 |
| BMI (kg/m^2^) | 0.69(0.59–0.81) | < 0.001 |
| Diabetes mellitus | 2.63(1.22–5.65) | 0.014 |
| Chronic kidney disease | 1.17(0.55–2.47) | 0.685 |
| Etiology | 0.83(0.48–1.44) | 0.515 |
| Child-Pugh B/C | 2.33(1.02–5.32) | 0.044 |
| MELD score | 1.10(0.96–1.26) | 0.157 |
| Total bilirubin (mg/dL) | 1.15(0.74–1.80) | 0.532 |
| Albumin (g/dL) | 0.53(0.28–1.01) | 0.054 |
| Prothrombin time INR | 0.65(0.04–10.6) | 0.764 |
| Creatinine (mg/dL) | 1.59(0.69–3.66) | 0.274 |
| eGFR (mL/min/1.73m^2^) | 0.99(0.97–1.01) | 0.347 |
| M2BPGi (C.O.I.) | 1.02(0.93–1.12) | 0.638 |
| Pentosidine (x10^2^) (μg/mL) | 1.10(1.02–1.19) | 0.012 |
| HCC | 2.09(0.87–5.01) | 0.100 |

BMI, body mass index; CI, confidence interval; C.O.I., cut-off index; eGFR, estimated glomerular filtration rate; HCC, hepatocellular carcinoma; INR, international normalized ratio; M2BPGi, Mac-2 binding protein glycosylation isomer; MELD, model for end-stage liver disease; OR, odds ratio.

**Table S2. Univariate analysis of factors associated with low gait speed**

| Variable | OR (95% CI) | *p* value |
| --- | --- | --- |
| Gender (Man) | 0.63(0.29–1.39) | 0.251 |
| Age (years) | 1.07(1.02–1.12) | 0.007 |
| BMI (kg/m^2^) | 0.93(0.85–1.02) | 0.132 |
| Diabetes mellitus | 1.95(0.88–4.31) | 0.099 |
| Chronic kidney disease | 1.69(0.76–3.76) | 0.202 |
| Etiology | 0.79(0.44–1.40) | 0.414 |
| Child-Pugh B/C | 4.03(1.71–9.46) | 0.001 |
| MELD score | 1.13(0.98–1.30) | 0.083 |
| Total bilirubin (mg/dL) | 1.20(0.76–1.89) | 0.433 |
| Albumin (g/dL) | 0.50(0.26–0.99) | 0.047 |
| Prothrombin time INR | 7.25(0.46–115.5) | 0.161 |
| Creatinine (mg/dL) | 1.29(0.55–3.05) | 0.560 |
| eGFR (mL/min/1.73m^2^) | 0.98(0.96–1.00) | 0.117 |
| M2BPGi (C.O.I.) | 1.04(0.95–1.14) | 0.401 |
| Pentosidine (x10^2^) (μg/mL) | 1.10(1.02–1.18) | 0.012 |
| HCC | 0.96(0.37–2.52) | 0.933 |

BMI, body mass index; CI, confidence interval; C.O.I., cut-off index; eGFR, estimated glomerular filtration rate; HCC, hepatocellular carcinoma; INR, international normalized ratio; M2BPGi, Mac-2 binding protein glycosylation isomer; MELD, model for end-stage liver disease; OR, odds ratio.

**Table S3. Univariate analysis of factors associated with mortality**

| Variable | HR (95% CI) | *p* value |
| --- | --- | --- |
| Gender (Man) | 1.16(0.55–2.43) | 0.702 |
| Age (years) | 1.01(0.97–1.05) | 0.599 |
| BMI (kg/m^2^) | 0.94(0.86–1.03) | 0.174 |
| Diabetes mellitus | 1.54(0.74–3.20) | 0.249 |
| Chronic kidney disease | 0.79(0.38–1.63) | 0.517 |
| Etiology | 1.20(0.69–2.07) | 0.522 |
| Child-Pugh B/C | 5.47(2.56–11.7) | < 0.001 |
| MELD score | 1.22(1.11–1.35) | < 0.001 |
| Creatinine (mg/dL) | 1.36(0.63–2.94) | 0.431 |
| eGFR (mL/min/1.73m^2^) | 1.00(0.98–1.02) | 0.840 |
| M2BPGi (C.O.I.) | 1.06(1.00–1.12) | 0.073 |
| Pentosidine (x10^2^) (μg/mL) | 1.09(1.06–1.12) | < 0.001 |
| Sarcopenia | 2.49(1.20–5.17) | 0.015 |
| Low gait speed | 3.62 (1.72–7.61) | < 0.001 |
| HCC | 4.69(2.22–9.90) | < 0.001 |

BMI, body mass index; CI, confidence interval; C.O.I., cut-off index; eGFR, estimated glomerular filtration rate; HR, hazard ratio; estimated glomerular filtration rate; HCC, hepatocellular carcinoma; INR, international normalized ratio; M2BPGi, Mac-2 binding protein glycosylation isomer; MELD, model for end-stage liver disease.
